# Metabolomics and Chemoinformatics in Agricultural Biotechnology Research: Complementary Probes in Unravelling New Metabolites for Crop Improvement

**DOI:** 10.3390/biology11081156

**Published:** 2022-08-01

**Authors:** Manamele Dannies Mashabela, Priscilla Masamba, Abidemi Paul Kappo

**Affiliations:** Department of Biochemistry, Faculty of Science, University of Johannesburg, Auckland Park Kingsway Campus, P.O. Box 524, Johannesburg 2006, South Africa; ngoatomd@gmail.com (M.D.M.); presh4u@rocketmail.com (P.M.)

**Keywords:** chemoinformatics, crop improvement, metabolic biomarker, metabolomics-assisted breeding, plant metabolomics

## Abstract

**Simple Summary:**

The world is facing an overarching threat to food security, particularly in developing nations. The issue is further exacerbated by the apparent impacts of biotic and abiotic stresses driving down crop yields and productivity. Conventional strategies to improve yields and sustain productivity have been employed, including plant breeding for favourable and resilient agronomic traits. However, the efficacy and success rates of these methods are declining, partly due to the rapid changes in climate variability and the emergence of new and resistant phytopathogens. Additionally, the process of creating new and improved transgenic varieties of crops is long and can be expensive. Thus, new and innovative technologies are required for crop improvement. This review explores recent advances in the science of metabolomics and chemoinformatics, which have presented an avenue for rapid and robust analysis; moreover, it explores the elucidation of the complex plant metabolome, providing the opportunity to decipher the reactionary mechanisms of plants to the surrounding environment through their metabolic activity. As such, specific metabolites can, thus, be selected as biomarkers for crop improvement based on their functional characteristics under varying environmental conditions (growth, development, and defence). This new knowledge can enhance breeding practices through rapid and robust metabolic engineering techniques for sustainable agriculture.

**Abstract:**

The United Nations (UN) estimate that the global population will reach 10 billion people by 2050. These projections have placed the agroeconomic industry under immense pressure to meet the growing demand for food and maintain global food security. However, factors associated with climate variability and the emergence of virulent plant pathogens and pests pose a considerable threat to meeting these demands. Advanced crop improvement strategies are required to circumvent the deleterious effects of biotic and abiotic stress and improve yields. Metabolomics is an emerging field in the omics pipeline and systems biology concerned with the quantitative and qualitative analysis of metabolites from a biological specimen under specified conditions. In the past few decades, metabolomics techniques have been extensively used to decipher and describe the metabolic networks associated with plant growth and development and the response and adaptation to biotic and abiotic stress. In recent years, metabolomics technologies, particularly plant metabolomics, have expanded to screening metabolic biomarkers for enhanced performance in yield and stress tolerance for metabolomics-assisted breeding. This review explores the recent advances in the application of metabolomics in agricultural biotechnology for biomarker discovery and the identification of new metabolites for crop improvement. We describe the basic plant metabolomics workflow, the essential analytical techniques, and the power of these combined analytical techniques with chemometrics and chemoinformatics tools. Furthermore, there are mentions of integrated omics systems for metabolomics-assisted breeding and of current applications.

## 1. Introduction

Food insecurity remains one of the most pressing environmental challenges of our time following decades of economic growth, technological advances, and agricultural intensification. Concerns about the various risks affecting food security are further exacerbated by the prospects of the ever-growing global population, which is projected to peak from 9.7 to 10 billion people by 2050 [1,2]. Additionally, the systemic risks related to food insecurity are linked to several concerns such as globalization, climate change, and sustainable agriculture. This includes diminishing natural resources such as arable land and water, overdependency on agrochemicals, and global demographics, including urbanization and diet preferences [3], all of which impact crop productivity, yields, and distributions. A culmination of systemic risks, thus, creates vulnerabilities and complexities in food systems that could lead to systemic crises such as food insecurity, particularly in underdeveloped and developing nations [3,4].

Climate change, much-aided by anthropogenic activities that include burning fossil fuels, mining, and industrial land and water pollution, induces extreme and frequent abiotic stressors such as drought, soil salinity, and metal toxicity. At the same time, the emergence of mutated, resistant, and more virulent plant pathogens and other biotic stressors has wreaked havoc on plant development and productivity, thus reducing yields by up to 80% [5,6]. Essentially, the impacts of biotic and abiotic stress on crop productivity have intensified pressure on the agroeconomic industry to develop sustainable methods for crop production to meet the growing demand. Plant breeding and genetic enhancement have since significantly contributed to meeting the needs of the human population at the beginning of the green revolution. Genetic techniques have led to the production of crops with favourable agronomic traits such as pest and disease resistance, drought tolerance, and increased yields [7,8]. However, crop breeding for elite genotypes has narrowed genetic and species diversity, generating vulnerabilities in bred crops to environmental and climatic changes. Breeders have been subjected to continual and direct intensive selection of better-performing crops, posing a severe challenge to further crop improvement [8].

Strategies to overcome these challenges include discovering novel variations by broadening the pool of genetic diversity and implementing the de novo creation or introgression of new crop varieties from studies of model crop plants [9]. On the other hand, the long labor-intensive and expensive process of generating genomic libraries and sequencing for phenotypes has deterred efforts to adopt the strategies mentioned above, causing a delay in the creation of resilient crop types [10,11,12]. Metabolomics studies have revolutionized the science of systems biology by enabling the full and accurate elucidation of an organism’s metabolic and cellular state as a reflection of the phenotype, thus providing a link to its trait and genotype [13]. Metabolomics refers to a holistic qualitative and quantitative analysis of all detectable metabolites (small molecules < 1500 Da) within a biological specimen (biological fluids, cells, and tissues) under specified exogenous and endogenous conditions at a specific time-point [14,15,16,17]. Plant metabolomics provided a new frontier in phytochemical and metabolite analysis to generate a comprehensive picture of plant–microbe interactions. Metabolomics presents a different spectrum of “-omics” technology. It is a data-driven and hypothesis-generating novel discipline. It combines advanced analytical techniques, multivariate data analysis tools, and modelling algorithms for comprehensive data analysis and representation [18,19]. As such, metabolomics offers unique avenues for deciphering complex metabolic mechanisms, understanding the phenotypic interpretations of perturbations in the omics pipeline of organisms, and generating a platform for biomarker discovery and identification in diagnostic studies [20,21]. This emerging technology has been useful in research fields including systems biology, medical sciences, and agriculture, representing a mature and robust technology in biological sciences. It is the closest and most accurate reflection of the phenotype at a molecular level and, thus, provides powerful tools for studying the phenotypic traits of plants.

Metabolomics studies apply advanced analytical tools such as gas chromatography–mass spectrometry (GC-MS), liquid chromatography–mass spectroscopy (LC-MS), and non-destructive nuclear magnetic-resonance spectroscopy (NMR) for the detection, identification, and evaluation of metabolites [21]. Additionally, high-resolution MS (HRMS), in combination with multivariate data analysis (MVDA) and statistical data analysis (chemometrics and chemoinformatics) tools, have further catapulted metabolomics to its position as the dominant analytical approach [18]. These tools and techniques have been used to decipher the biochemical nature of ecological events (systems biology), such as the effects of biotic and abiotic stress on plant growth and development. Additionally, plant metabolomics approaches have enabled scientists to uncover the metabolic responses and adaptations of plants to both biotic and abiotic environmental factors. The advantage of metabolic biomarker discovery and identification has opened doors for the screening and selecting of elite crop varieties. At the same time, the integration of metabolomics and other omics techniques presents exciting opportunities for agricultural biotechnology and crop improvement.

This review highlights the potential for metabolomics in the agroeconomic industry and improves crop research. We describe advances in analytical techniques, chemometrics, and chemoinformatics tools and the standardized workflow for plant metabolomics research. We also discuss the advantages of plant metabolomics studies in elucidating biotic and abiotic stress responses in plants, their adaptation to stress conditions and the prospects of the integrated omics pipeline in agricultural biotechnology and for metabolomics-assisted breeding and crop improvement.

## 2. Metabolomics and Chemoinformatics Tools as Prospects for Crop Improvement

**Figure 1:**
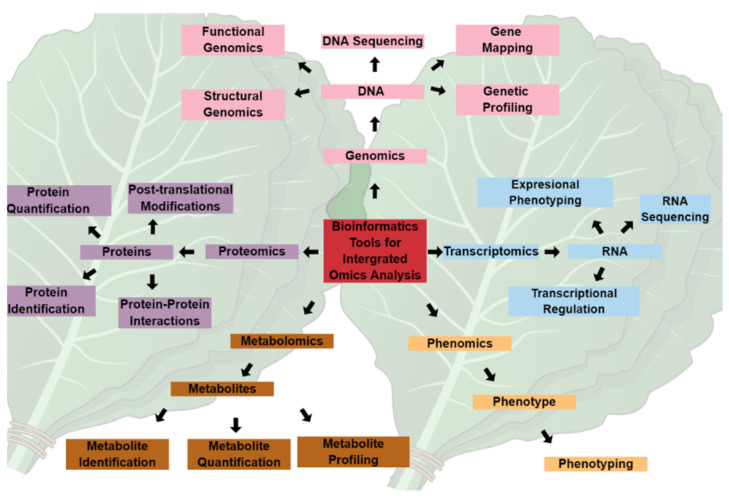
The central dogma of biology and integrated omics technology. Systems biology stems from various aspects of the central dogma making up the omics sphere, which is inclusive of genomics, transcriptomics, proteomics, metabolomics, and phenomics. Dedicated applications of individual omics fields have been useful in the elucidation of plants and their interactions with the surrounding environment. The integration of these fields, in combination with bioinformatic and chemoinformatics tools, can revolutionize the understanding of the underlying mechanisms of plant responses to environmental conditions. The massive data generated can be used in targeted gene editing, recombinant DNA technology, protein synthesis, and metabolite engineering for crop improvement and sustainable agriculture.

Omics technologies, i.e., genomics, transcriptomics, proteomics, and metabolomics, illustrated in Figure 1, have provided holistic approaches to studying and understanding the biology and physiology of living organisms. However, these various omics approaches have primarily been used in isolation, and the data generated are rarely integrated across the omics spectrum, particularly in biomarker identification for crop improvement. For instance, genomics-assisted breeding has revolutionized the agroeconomic industry over the past few decades by generating climate-tolerant and biotic-resistant crop varieties [22]. Nevertheless, the expression of phenotypic traits is regulated at a metabolic level, which requires a better understanding of the small molecules and their interactions in facilitating the metabolic activities linking gene expression to the observed phenotype. Therefore, linking metabolomic and genomic data and integrating the omics techniques could revolutionize the discovery and identification of biomarkers, representing the central dogma of biology.

### 2.1. Plant Metabolomics

Plants are some of the most chemically diverse living organisms. The plant kingdom consists of approximately one million different metabolites, and each plant can produce 5–25,000 compounds, with the vast majority still unknown [9,23]. Due to their sessile nature, plants produce many compounds and metabolites to aid their unique adaptive features. They have been distinctively classified into two major groups, namely primary and secondary metabolites, based on their functional properties in plant growth, development, and survival [23]. These metabolites are also interlinked through a series of highly complex metabolic pathways. Primary metabolites are involved in the biosynthesis of sugars, lipids, amino acids, and TCA intermediates such as organic acids. They are essential for plant growth and development as they mediate the TCA cycle, glycolysis, and photosynthesis. According to Razzaq and colleagues [24], alteration in the primary metabolism of plants can lead to photosynthetic malfunction and osmotic imbalance. These challenges can be mitigated by secondary metabolites’ antioxidant and osmoregulatory capabilities. Plants also produce specialized secondary metabolites such as phenolics, alkaloids, and terpenoids, which alleviate biotic and abiotic stress susceptibility, some of which have been identified as unique biomarkers for plant performance under various environmental conditions earmarked for crop improvement programs [24].

Therefore, unravelling the unique metabolic features responsible for plant survival under varying stress conditions such as drought, salinity, metal toxicity, or even pest and pathogenic attacks for metabolic engineering in crop improvement is essential. Plant metabolomics investigates the mechanistic nature of plant responses to biotic and abiotic stress from a metabolic viewpoint and the types of metabolites involved [25]. Knowledge of these processes could help predict physiological traits and how a plant can be engineered at a metabolic level to better respond to its environment. This revelation presents metabolomics as a suitable tool for biomarker discovery and identification for plant breeding [26]. Additionally, plant metabolomics can help identify and link genes associated with crop quality, productivity, yields, and survival under adverse environmental conditions, particularly under persistent threats of climate change.

Furthermore, metabolomics represents the downstream measure of the functional state of the cell, which is immeasurable at any other omics level (genomic, transcriptomic, and proteomic) [24,27]. Therefore, metabolite profiling provides a snapshot of the real-time state of the plant. Combining metabolomics techniques with state-of-the-art chemoinformatics and chemometrics statistical and analytical tools positions plant metabolomics as a powerful tool for elucidating plant biochemistry research to facilitate crop improvement.

### 2.2. Analytical Tools and Approaches in Plant Metabolomics Research

The elucidation of a plant’s metabolome provides an insight into the biochemical and regulatory processes occurring during plant metabolism. This process can be quantitative, which applies a targeted metabolomics approach concerned with the quantification and identification of a set of known target classes or groups of metabolites from a biological specimen. On the other hand, untargeted metabolomics focuses on the detection and qualitative and semi-quantitative global profiling of known and unknown metabolites, providing comprehensive metabolome coverage [27,28]. Both targeted and untargeted metabolomics approaches have their own inherent advantages and disadvantages. For instance, targeted analysis provides absolute quantification of samples, and it has better sensitivity, selectivity, and higher accuracy; however, it is not comprehensive and, thus, leads to the detection of fewer metabolites, which increases the risk of overlooking metabolic responses of interest [14,19]. An untargeted metabolomics approach offers comprehensive coverage of the metabolome; it is the most commonly used approach, partly due to the opportunity for biomarker discovery, and provides high throughput. In contrast, untargeted metabolomics is semi-quantitative and generates large quantities of data made up of unknown compounds, which can be difficult to annotate [24,25,27]. The current review focuses on the application of untargeted metabolomics for crop improvement strategies. Plant metabolomics analysis employs advanced, high-throughput separatory techniques such as liquid or gas chromatography (L/GC), instrumentation for mass spectrometry (MS) is frequently hyphenated, as well as nuclear magnetic resonance (NMR) for the detection, identification, and evaluation of the complex plant metabolome [17,29].

LCMS and GCMS are the two most-utilized techniques due to their high sensitivity, selectivity, robustness, and reproducibility. The selective nature and high resolution provided by high-performance liquid chromatography (HPLC) and ultra-high-performance liquid chromatography (UHPLC), combined with proficient mass spectrometry, have made these techniques the gold standard for metabolomics studies, and for metabolite profiling and analysis [30]. Moreover, LCMS-based metabolite profiling is compatible with higher-molecular-weight, polar, and thermo-labile compounds, including secondary metabolites such as phenolics, vitamins, and glucosinolates [24,25]. In contrast, GCMS is beneficial for the detection of volatile and thermally unstable compounds and is suitable for primary metabolites, including organic acids, amino acids, sugars, and low-molecular-weight hydrocarbons, which generally require chemical derivatization steps during sample preparation [24,25]. On the other hand, NMR provides information on the structural units of unknown metabolites, high accuracy, small sample volumes, and, thus, ease of sample preparation [19,31] Furthermore, it is non-destructive and requires no hyphenation to be separatory, or chromatographic techniques. However, this technique falls short in resolution and sensitivity, resulting in a lower plant metabolome coverage than MS. Nevertheless, NMR-based metabolomics is a convenient, quick, and highly efficient tool for plant metabolomics in mapping biological pathways and identifying similar samples [24,32].

The choice of a metabolomics approach is generally determined by the analytical tool used based on its accuracy, selectivity, and sensitivity or the sample and target metabolites (polar/nonpolar, volatility) under investigation. Other metabolomics techniques include high-performance thin-layer chromatography (HPTLC), direct-infusion mass spectrometry (DIMS), Fourier transform ion-cyclotron resonance mass spectrometry (FI-ICR-MS), and capillary electrophoresis–mass spectrometry (CE-MS) [24,25,33,34]. Applying these techniques has made it possible to monitor plant responses and metabolic reprogramming under biotic [35,36] and abiotic stress [37,38], as further discussed in plant metabolomics applications for crop improvement strategies.

### 2.3. An Overview of the Standardized Workflow for Plant Metabolomics Studies

Metabolomics studies generate large quantities of phytochemical data, which have, thus far, contributed to the curation of metabolic and metabolite databases. The data acquired are generated from different laboratory investigations, objectives, study designs and data-acquisition methods (extraction procedures and instrumentation). Plant metabolomics studies rely upon previously generated metabolomics databases to annotate and identify metabolites; therefore, a standardized metabolomics approach is highly recommended to ensure principal data, reproducibility, and the proper interpretation of experimental data for adequate results. This approach guarantees that metabolite entries in databases and the published literature are accurate and error-free. Quality control (QC) and quality assurance (QA) measures are, thus, needed during the experimental design to formulate a standardized operating procedure (SOP) and to ensure high quality of the data acquired in plant metabolomics [27,28,39]. Earlier recommendations and proposals have been made, such as the Metabolomics Standards Initiative (MSI) and the Architecture for Metabolomics (ArMet) [39,40,41], which provide guidelines on the adoption of standard metabolomics in sampling, extraction, instrumentation, quantification, metabolite identification, documenting novelties (important for biomarker discovery), and checklists for QA and QC to promote principled curation and transmission of metabolomics data.

Furthermore, general metabolomics workflows for both targeted and untargeted plant metabolomics have been presented, as discussed by Tugizimana et al. [14], Nephali et al. [31], and Hamany Djande et al. [42]. These include (1) experimental design, (2) sample preparation, (3) data acquisition, (4) data mining and processing, and (5) statistical analysis and biological interpretation, as briefly described below. As described by the metabolomics workflow, the outcome of the experimental design should be guided by a clear, concise, and specific biological question to maximize metabolomics outputs and avoid the misinterpretation of data [31,43].

#### 2.3.1. Sample Preparation: Harvesting and Metabolite Extraction

Sample preparation forms an integral part of plant metabolomics studies. With various harvesting, handling, and extraction procedures; different sample materials in the form of leaves, roots, or stems; and a range of analytical platforms, great care should be taken at this stage of the experimental procedure as significant biases and technical variations can impact the results. Unfortunately, even modern analytical methods cannot compensate for improper sample preparation; therefore, an altered metabolome will foil the comprehensive profiling of the plant metabolome [43]. Additionally, it is essential to maintain the integrity of the samples and preserve the metabolic content. Slight variations in the immediate environmental conditions of the source material such as wounding, shade, or exposure to chemicals can induce enzymatic reactions that metabolize compounds and cause metabolic perturbations, influencing analysis outcomes. Usually, cryogenics is used to quench plant material by submerging the samples in liquid nitrogen to prevent further metabolic activity [24,43]. Sample material can be stored frozen (−20/80 °C) or freeze-dried and lyophilized at room temperature until extraction. However, Martins and co-workers [43] and Kim and Verpoorte [44] suggest using fresh plant material to quantify semi-volatile and volatile compounds.

Several extraction protocols have been developed and applied in metabolomics studies based on the aforementioned factors. The chosen method of sample preparation and extraction determines the types of compounds to be extracted or detected and is mostly dictated by the analytical platform to be used [19]. This method is generally followed by pre-analytical sample preparation, including sample concentration, purification, and derivatization [14,19]. A suitable extraction procedure will ensure high recovery percentages of metabolites from sample tissue. However, as previously mentioned, the plant metabolome is highly diverse and complex, consisting of thousands of different metabolites varying in polarity, structure, chemical behaviour, volatility, and solubility; therefore, complete metabolome coverage is difficult [14,31]. Researchers have performed method optimization studies testing the extraction efficiency of various organic solvents such as ethanol and methanol at different concentrations [45,46] to extract polar and nonpolar metabolites. Even so, extraction of the entire plant metabolome is near impossible.

Nevertheless, with the innovations and technological advances in metabolomics, many extraction protocols have been developed to mitigate the loss of metabolic data and increase extraction efficiencies, as reviewed by Razzaq and co-workers [24]. These techniques include solid-phase microextraction (SPME) and ultrasound-assisted extraction (UAE), most commonly used for plant metabolomics. Microwave-assisted extraction (MAE) and laser microdissection (LMD) are new techniques that allow high-throughput metabolite extraction with high speed and accuracy; LMD is also suitable for the extraction of metabolites from microscopic samples [24,47,48]. However, some of these extraction procedures can be expensive or time-consuming, indicating the continuous innovation and development required to streamline and standardize plant metabolomics studies. Meticulous sample preparation is followed by metabolomics data acquisition using analytical metabolomics-based techniques and chromatography (discussed in Section 2.2). Like the scrupulous harvesting and precise sample extraction, careful consideration of the analytical platform’s performance (repeatability and reproducibility) should be taken to ensure the accuracy and high quality of the generated data [43]. Generally, QC samples are prepared to evaluate the reliability and reproducibility of the analytical instrument; these can be a pooled mixture of all the samples under investigation and authentic reference compounds.

#### 2.3.2. Chemometrics and Chemoinformatics Tools for Metabolite Annotation and Biomarker Identification

Biomarker identification forms an integral part of plant metabolomics, particularly when investigating a plant’s adaptive or response mechanisms to varied environmental conditions. Untargeted whole metabolome coverage presents the enormous task of data deconvolution. The complexity and diversity of the plant metabolome further complicate the analysis of raw data from extracted metabolites representing various metabolic activities in the plants under investigation. The post-analytical step in the metabolomics workflow requires powerful and sophisticated chemometrics and chemoinformatics (also called cheminformatics) tools for data mining, pre-processing, pre-treatment, and statistical modelling using machine-learning (ML) algorithms, followed by metabolite or biomarker annotation and identification, and biological interpretation [14,31,49]. Chemometrics and chemoinformatics, often used interchangeably, are computational data analysis tools designed to deal with massive amounts of metabolomics data. Chemometrics is a multivariate method that employs mathematical modelling and statistics to mine and process, and obtains relevant chemical information from biological or chemical systems from multivariate data. In relation to this, chemoinformatics is concerned with collecting and utilizing chemical data derived from modelled chemometrics data in metabolomics studies to predict the biological behaviours of known and unknown compounds in silico [50,51]. The most-used chemometrics tool in metabolomics studies is multivariate data analysis (MVDA) for accurate comparative evaluation of the quantitative or qualitative metabolic features representing the phenotypes of plants [41]. MVDA methods are useful in reducing the dimensionality of the data and extracting the maximum information from several thousand processed variables, leading to potential biomarker discovery [52,53].

The two most common chemometrics approaches (unsupervised and supervised MVDA methods) are generally used to study raw metabolomics data. Unsupervised methods such as principal component analysis (PCA) deconvolute complex metabolomic data, subsequently summarising global data by revealing underlying data patterns that reflect experimental or biological variables [53,54]. PCA can further analyze data quality by identifying outliers or hidden biases from a study. In plant metabolomics, PCA score plots reveal the variations in plants with differential metabolite profiles based on group separations. However, PCA is an experimental technique and does not account for class-based separations due to its unsupervised nature. Hence, a supervised approach such as partial-least-squares discriminant analysis (PLS-DA) is employed to account for class-based separations. PLS-DA and its extensions such as orthogonal PLS-DA (OPLS-DA) and sparse PLS-DA (sPLS-DA) describe the most significant variance to differentiate between practical classes to decipher the metabolic features that are most significant to the observed classification [52,55]. OPLS-DA is a binary classifier that uses two experimental group systems to compare metabolic features (generally treated vs. untreated samples); from this, metabolites with an enormous impact on the projection are selected and termed significant metabolic markers responsible for the differences in metabolic features from the two experimental groups under investigation [56,57].

The significant metabolites are generally represented as *m*/*z* features or identities (IDs), and, thus, require the process of metabolite annotation and identification. The identification of metabolites is the ultimate goal of untargeted metabolomics studies, a crucial step in the discovery of new metabolites and in the elucidation of the metabolic activities of plants in response to the changes in their immediate environment [17,19]. The putative annotation and identification of metabolites usually depend on the acquisition of sufficient and accurate structural information including accurate mass measurement and fragmentation patterns; this is followed by the calculation of empirical formulae corresponding to the measured accurate mass and elemental composition. Finally, matched spectral data are compared to curated metabolite databases and the published literature to identify the compound of interest [14,19,42]. These significant metabolic markers are further investigated to determine their biological significance, impact on the plant’s primary or secondary metabolism for plant growth and development, resistance, and tolerance to biotic and abiotic stress using chemoinformatics tools. This process can often lead to discovering and selecting novel metabolic features or new metabolites essential in plant growth or defence based on set metabolites’ up-/down-regulation under specific conditions, and is potentially valuable for crop improvement. There are some freely available chemometrics tools for data storage, processing, and annotation, such as XCMS (https://xcmsonline.scripps.edu, accessed on 12 March 2022), MAVEN, Mzine, MetaboAnalyst (also a chemoinformatics platform), and MS-Dial (http://prime.psc.riken.jp/Metabolomics_Software/MS-DIAL/, accessed on 12 March 2022) [19,58]. Commercially available chemometrics tools include Markerlynx, SIMCA (http://umetrics.com/products/simca, accessed on 12 March 2022), and Matlab, subscription-based software, or online platforms [19,24].

Chemoinformatics, an extension of chemometrics tools in metabolomics studies, is a discipline applied to decipher the functional information and properties of chemicals and metabolites (selected metabolic markers or new metabolite discoveries), the conformational analysis of metabolites, the assessment of intermolecular interactions, pathway analyses, and the biological interpretation of metabolomics data [50,59]. In plant metabolomics studies, chemoinformatics tools are helpful in data visualization and interpretation. Following metabolite marker identification and annotation using chemometrics, the metabolites with statistical significance are further investigated and mapped into metabolic or biochemical pathways. PubChem (https://pubchem.ncbi.nlm.nih.gov/, accessed on 20 March 2022) is the largest chemical information database, housing approximately 111 million compounds. It is helpful in metabolite annotation and provides information on these molecules’ physical properties and biological activity. PubChem is further linked to more than 800 other data sources containing curated or highly annotated bioactive compounds such as HMDB (https://hmdb.ca/, accessed on 20 March 2022), ChEBI (https://www.ebi.ac.uk/chebi/, accessed on 8 April 2022), and KEGG (http://www.genome.jp/kegg/, accessed on 10 April.2022); these include detailed information on the compounds’ associated biological pathways, macromolecular targets, mechanisms of action, biological effects, disease associations, toxicological data, and pharmacogenomic consequences [60,61]. This curated information is essential for assigning bioactivity and biological roles to identified metabolic markers in plants under varying environmental conditions, and has the predictive advantage of the adaptability of plants consisting of such metabolic markers (or lack thereof) under specified conditions. In the last decade, MetaboAnalyst (https://www.metaboanalyst.ca/, accessed on 7 May 2020) has been developed as the gold standard for comprehensive plant metabolomics data analysis and interpretation.

Most importantly, it provides tools for biomarker selection via ROC (receiver operating characteristic), which the platform further incorporates into the in-house MetPA (metabolic pathway analysis) domain (http://metpa.metabolomics.ca, accessed on 19 May 2022) [62,63]. Such chemometrics and chemoinformatics tools have revolutionized research studies for crop improvement and metabolomic-assisted plant breeding. Their application in plant metabolomics can provide insights into the identification of metabolic markers and novel metabolites to predict crop behaviour under biotic/abiotic stress, breeding for high yields and crop productivity, and climate-smart or highly adaptable crop varieties.

## 3. Current Applications of Plant Metabolomics in Crop Improvement

In recent years, metabolomics has emerged as a promising tool to unravel the underlying mechanisms in plant responses to both biotic and abiotic stress conditions and understand how these mechanisms can form the basis for application in crop improvement by selecting the metabolic markers representing desired performance traits such as resistance, tolerance, and plant growth in terms of both productivity and yield. Given the severe impact of biotic and abiotic stress on crop production and output, this review further discusses the application of plant metabolomics to improve crop adaptability to the stress conditions previously mentioned. We explore studies investigating metabolic marker identification for abiotic-stress tolerance and biotic-stress resistance, and advances in crop improvement applications.

### 3.1. Examples of Metabolomics for the Elucidation of Plant-Growth Promotion

Plant growth and development are facilitated by many metabolic events spanning several metabolic pathways and affecting many physiological processes. The primary metabolism forms the basis of energy and biomass production in plants from derived photosynthates (sugars), lipids, and other macromolecules. Thus, metabolomics profiling can provide insights into the mechanistic nature of plant growth, development, and subsequent yield quality to further reveal the metabolites most suitable for enhancing these processes in plants. Nephali and co-workers [37] observed a decrease in alanine, serine, aspartic acid, cysteine, proline, and threonine in maize plants treated with plant-growth-promoting rhizobacteria (PGPR) using ultra-high-performance liquid chromatography–high-definition mass spectrometry (UHPLC-HDMS) analysis and chemometrics and chemoinformatics tools such as PCA, OPLS-DA, SIMCA, and MetaboAnalyst. The study revealed the redirection of amino acids towards energy production, feeding into the tricarboxylic acid (TCA) cycle and protein biosynthesis for increased plant biomass and, thus, growth promotion. The UHPLC-MS/MS profiling of plant growth-promoting metabolites in the rhizosphere of maize plants showed the differential accumulation of amino acids, amino acid derivatives, and other different metabolic compositions, resulting in improved root and shoot length [64]. This study used PLS-DA to determine and select significant differential metabolites (potential metabolic markers) matched against the KEGG database for further investigation.

Furthermore, Othibeng and colleagues [38] reported an increase in phytohormones such as indole-3-acetic acid (IAA) and salicylic acid (SA), which are responsible for plant regulatory processes such as seed germination and seedling growth, using an untargeted metabolomics approach and chemoinformatics tools and databases such as PubChem, Chemspider, and the Dictionary of Natural Products for metabolite annotation. The studies mentioned above applied the advantages of metabolomics technologies, combined with chemometrics and chemoinformatics tools, to probe the roles of essential metabolites in plant growth and development. This approach presents a critical frontier in elucidating the metabolic features governing plant metabolism, as further discussed below.

### 3.2. Elucidation of Plant Response to Biotic and Abiotic Stress

**Figure 2:**
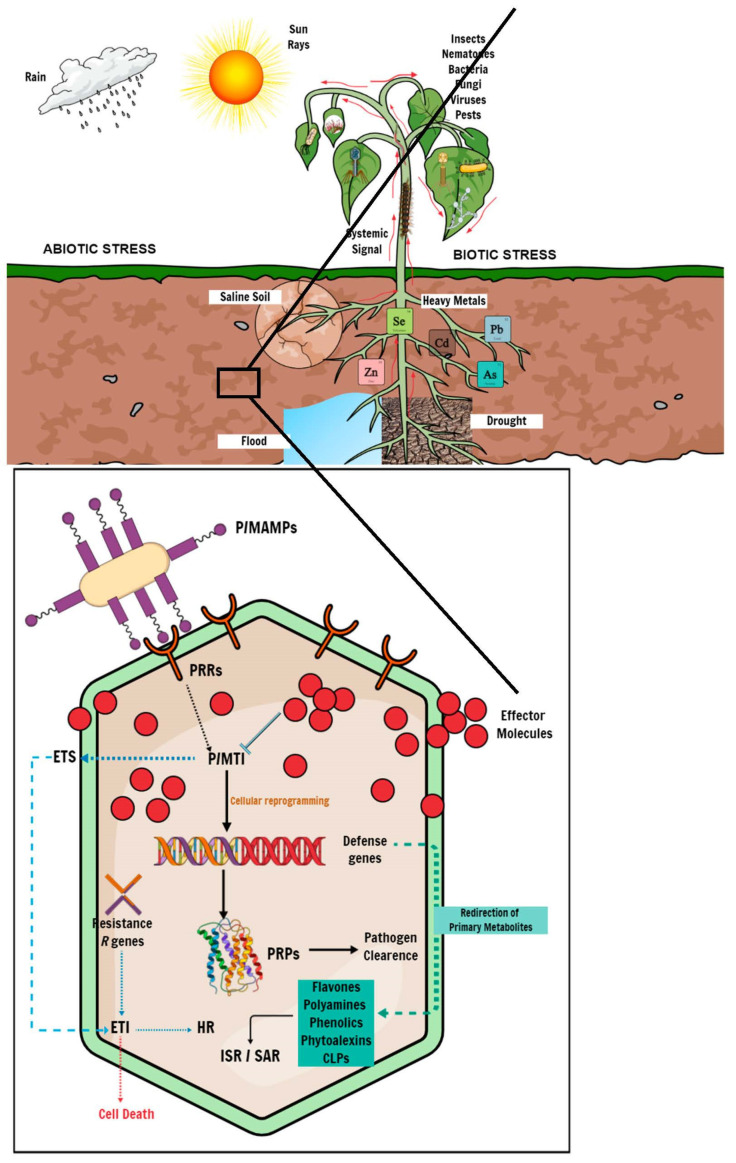
Schematic representation of plant–pathogen interactions. Plants induce a cascade of genomic and metabolic processes to defend against invading pathogens. At the onset of plant–pathogen interaction, the host plant perceives the invading microbe using PRRs, which trigger the first line of intracellular defence called P/MTI. P/MTI-mediated response leads to the production of antimicrobial compounds and PRPs that inhibit pathogen proliferation. In response, the invading pathogen produces effector molecules to counter the PRPs, thus inducing ETS to suppress P/MTI and establish a disease state. Host plants ultimately deploy effector-triggered immunity because of ETS, which leads to localized cell death through an HR mechanism to limit disease progression. Distressed plants further use systemic signaling to communicate stress conditions to unaffected parts of the plant to induce and mount a defence mechanism before a challenge from either biotic or abiotic stress in a phenomenon called ISR, or an alternative SAR, in response to abiotic stress. In addition to systemic-defence gene expression, the plant defence response involves several metabolic pathways spanning the primary and secondary metabolism, leading to metabolic reprogramming. Under stress conditions, host plants redirect products of the primary metabolism, such as amino acids and organic acids, to precursors of the secondary metabolism for the biosynthesis of specialized metabolites, including phenylpropanoids, flavonoids and phytoalexins.

Plants are constantly exposed to the elements during their developmental cycle and have, thus, evolved complex mechanisms and adaptive measures to combat biotic and abiotic stress. Biotic stressors come in the form of diseases caused by living organisms such as viruses, bacteria, fungal pathogens, and pests. At the same time, abiotic stresses include, but are not limited to drought stress, soil salinity, and environmental contaminants such as heavy-metal toxicity. Plants lack specialized immune cells to establish and mobilize an adaptive immune response to either biotic or abiotic stress. However, plants can acquire immunity upon exposure to certain biotic or abiotic stimuli, which leads to the activation of enhanced inducible defence mechanisms in anticipation of subsequent antagonistic attacks [65]. Exposure to an environmental stimulus induces a metabolic response and triggers defence strategies through the activation of defence genes, followed by metabolite perturbations, i.e., the redirection of primary metabolites to substrates of secondary metabolism to produce specialized secondary defence metabolites for adaptation to the stress (Figure 2) [66,67]. It is, thus, essential to understand the biochemical mechanisms underlying the plant defence response, identify the phenotypic and metabolic features responsible for the biotic and abiotic stress response, and screen for resistant and tolerant cultivars. This information would help with future genetic and metabolic engineering for crop improvement.

#### 3.2.1. Adaptation to Biotic Stress

Ordinarily, plants employ innate physiological defence barriers, including waxes, reinforced cell walls, lignin deposition, and readily available antimicrobial compounds or enzymes (phytoanticipins) to prevent pathogen infections. Upon successfully breaching the primary defence barriers, plants use signaling compounds such as pathogen-recognition receptors (PRRs), localized as transmembrane receptors or other subcellular compartments, to recognize molecular features from the pathogen microbe called pathogen-/microbe-associated molecular patterns (P/MAMPs). The perception of P/MAMPs activates a cascade of signaling mechanisms to induce a secondary defence system called P/MAMP-triggered immunity (P/MTI) [19,31,68]. P/MTI activation results in cellular reprogramming, including the activation of defence genes and the subsequent production of antimicrobial compounds such as phytoalexins or pathogenesis-related proteins (PRPs). Ultimately, P/MTI leads to pathogen clearance in the plant. However, pathogens have evolved mechanisms to suppress P/MTI by producing virulent effector molecules that induce effector-triggered susceptibility (ETS) in the plant, potentially causing a disease state [69]. To mitigate the effects of ETS, plants mount an immune response mediated by the activation of resistance (*R*) genes encoding specialized *R* proteins, which recognize pathogen-specific effector molecules and elicit effector-triggered immunity (ETI). The initiation of ETI results in a localized hypersensitive response (HR), immediately leading to cell death around the site of infection, thus preventing disease progression throughout the plant (Figure 2) [70,71].

Several metabolomics studies have reported the metabolic changes occurring in plants due to plant–microbe interactions and the metabolic reconfigurations for adaptation to pathogenic attacks. In a study conducted on UHPLC-ESI-qTOF-MS and UHPLC-QqQ-MS, the profiling and quantification of amino acids and phytohormones revealed differential reprogramming in the primary metabolism of *Phytophthora capsica*-infected tomato plants [72]. Reprogrammed metabolites belonged to the major classes of flavonoids, fatty acids, amino acids, TCA intermediates, glycoalkaloids, and HCA derivatives, indicating a complete metabolome response of the plants to pathogenic infection. In a separate study, using similar investigative tools, Mhlongo et al. [36] revealed tissue-specific defence responses of tomato plants to pathogenic infection. The study showed that roots, stems, and leaves undergo differential metabolic changes in response to biotic stress challenges to ward off infections in one part of the plant. This mechanism helps prevent further disease progression to other parts of the plant, a phenomenon known as systemic acquired resistance (SAR), suggesting that plant defence mechanisms can be as tissue-specific as they are species-specific.

Metabolomic profiling of tomato plants responding to *Ralstonia solanacearum* was performed to detect metabolites used as candidate biomarkers associated with the defence response against *R. solanacearum* infection [35]. These included hydroxybenzoic acids (HBAs), hydroxycinnamic acids (HCAs), phenolic acids, and flavonoids, which play pivotal roles in plant defence. For instance, elevated levels of the HBA salicylic acid were reported in the tomato plants following *R. solanacearum* infection, which is consistent with their function as stress-signaling compounds for defence-response activation through SAR. Phenylpropanoids are essential for lignin biosynthesis and cell-wall strengthening. These compounds, thus, assist plants in formulating a solid cell-wall defence to ward off pathogenic attacks [73].

Recently, Cuperlovic-Culf and co-workers [33] used NMR-based metabolomics analysis to discover unique biomarkers for disease resistance against *Fusarium graminearum* in the wheat metabolome. The study revealed an accumulation of identified defence-related metabolites such as trehalose, asparagine, phenylalanine, myoinositol, 3-hydroxybutyrate, and L-alanine as biomarkers for disease resistance. It was concluded that the identified unique metabolic markers could provide breeders with reliable predictions of resistance or susceptibility to certain diseases, which would be helpful in metabolic engineering for induced resistance in crop plants. Early metabolomic studies also showed the potential of metabolomics to study biochemical changes in plants in response to their immediate environment. For instance, Jones and colleagues [74] showed *Magnaporthe grisea*-induced metabolic changes in the rice metabolome in an LC-MS-, GC-MS-, and NMR-based metabolomics study. According to the authors, the study demonstrated the strength of metabolomics in evaluating the overall effects of pathogen infection on plants. Other studies elucidated the response mechanisms of plants to insect attacks, as reviewed by Razzaq and co-workers [24]. Liu and colleagues [75], and Shavit and co-workers [76], studied the resistance of rice and wheat against *Chilo suppressalis* and aphids, respectively. Metabolomics analysis showed the activation of defence-related phytohormone, and interpenoid-related and shikimate-mediated secondary metabolism in rice responding to *C. suppressalis* feeding. At the same time, a significant induction of benzoxazoles was observed in wheat genotypes subjected to aphid feeding. Both studies reveal the diverse metabolic response of plants under varying environmental conditions. The differential accumulation of these metabolites highlights aspects of a plant defence response’s biochemical and physiological approaches; some of these metabolites can further be investigated as biomarkers associated with biotic stress. Furthermore, such metabolites can be programmed for higher production or applied to enhance plant defence.

#### 3.2.2. Adaptation to (Selected) Abiotic Stress

Abiotic stresses are the most prominent and significant limiting factors in crop production; they result from the extended exposure of plants to extreme environmental conditions such as drought, floods, soil salinity, metal toxicity from acid soils, extreme cold or hot temperatures, and often, nutrient starvation. Plants produce specialized metabolites that regulate metabolic and physiological changes caused by the abovementioned stressors to restore homeostasis and maintain environmental adaptability [77]. Metabolomics studies have been explored to better understand the metabolic regulatory mechanisms employed by plants in response to abiotic stress. Recently, Othibeng and colleagues [38] evaluated metabolic reprogramming in maize plants in response to nutrient starvation. A UHPLC-qTOF-MS analysis revealed a significant reduction in amino acids; reduced proline, serine, aspartic acid, threonine, phenylalanine, alanine, and tryptophan were observed in nutrient-starved plants compared to the control. Significant reductions in components of the phenylpropanoid pathway and flavonoid biosynthesis were observed. Similar results were reported from metabolomic profiling by Sung and co-workers [78]. They found decreased levels of amino acids in the leaves of tomato plants due to nitrogen, potassium, and phosphorous deficiency.

Drought and salinity stress are two of the most-studied environmental stresses affecting crop production and yields. Plants subjected to drought stress are generally affected due to limited water uptake from the roots or excessive transpiration rates from high temperatures [25]. Current trends in climate change are expected to exacerbate the impacts of drought on global crop productivity due to increased temperature and unstable rainfalls, which could increase drought severity by 20% [25,79]. Metabolomics profiling of crops and model plants has been performed to elucidate plant responses to drought conditions, evaluate plant adaptations to drought stress, and discover metabolic biomarkers for stress tolerance. For instance, GC-MS analysis of barley (*Hordeum vulgare* L.) exposed to drought conditions showed an accumulation of amino acids (proline, glycine, valine, threonine, isoleucine, phenylalanine), sugars (fructose, sucrose, glucose) and organic acids such as malate [80]. Proline was the most significantly accumulated metabolite, suggesting the importance of this amino acid in the drought response. Proline functions as an energy source, a stress-signaling molecule, and a ROS-scavenging osmolyte [80,81], while phenylalanine serves as a precursor to producing specialized secondary defence metabolites through the phenylpropanoid pathway. Thus, applying these amino acids, particularly in formulations, can help facilitate crop improvement for biotic-stress tolerance, further improving crop quality, productivity, and yields.

Additionally, Kang and colleagues [30] used metabolomics to evaluate differential changes in the metabolic states of wheat genotypes to cope with drought stress. The study revealed that roots and shoots were metabolically activated to enhance water and nutrient uptake, as indicated by the significant increase in amino acids and sugars in the roots of drought-tolerant genotypes, thus maintaining growth and productivity during the drought stress conditions. Furthermore, several metabolomics studies, with the aid of both GC- and LC-MS analytical techniques, have reported the differential accumulation of amino acids, organic acids, and sugars as the primary metabolic adjustment in drought-stricken plants [82,83,84,85]; this can be an indication of the importance of these metabolites as the first line of defence in plant responses for drought tolerance.

Differential accumulation of secondary metabolites also has been reported in plants responding to drought stress. Recently, an LC–electrospray ionisation–triple quadrupole–linear ion trap (QTRAP)-MS was performed to evaluate the metabolic responses of *Dendrobium sinense*. An increase in flavonoids, alkaloids and phenylpropanoids was recorded under drought stress [86]. Another recent study reported the reprogramming of the secondary metabolism reflected by differential accumulations of HCAs, HCA derivatives, and flavonoids in maize plants under drought stress [37]. Furthermore, a series of phenolics, alkaloids, and flavonoids were also accumulated in wheat genotypes exposed to drought conditions [87]. Interestingly, these metabolites were higher in the drought-tolerant genotype than in the drought-sensitive genotype, indicating the vital roles of the secondary metabolites in drought tolerance capacity. The antioxidant properties of secondary metabolites can prevent the accumulation of ROSs in plants, reducing damage to cell membranes, protein degradation, and enzyme inactivation, and, thus, improving drought tolerance [86,87].

On the other hand, the impacts of salinity stress on crops and plants have been extensively investigated. Salinity stress causes osmotic imbalance and oxidative stress, which is induced by ion toxicity and unstable ion uptake in plants; this leads to interruptions in water and nutrient uptake, reduced growth rates and photosynthetic capacity [77,87,88]. The strategies for mitigating the impacts of salinity stress in plants span the primary and secondary metabolism, reflecting the basal work conducted at the genome level. The primary metabolism is pivotal in regulating salinity stress responses; essential roles have been suggested, including energy production for the initial cost of stress-related metabolic response mechanisms, the activation and regulation of signaling cascades, and the provision of substrates for secondary metabolism [57]. In contrast, the secondary metabolism provides specialized metabolites that facilitate the salinity stress response through such mechanisms as antioxidant synthesis pathways for ROS scavenging and the reduction of oxidative damage [57,86]. A recent Fourier transform mass spectrometry LTQ orbitrap (FTMS)-based metabolomics study by Sarri and colleagues [89] found that secondary metabolites from saponins and hydroxycinnamic acids play a significant role in increased salinity-stress tolerance in *Medicago sativa* and *Medicago arborea* species. Similar findings were reported by Cai and co-workers [90], who found differential accumulation of secondary metabolites in salt-stressed plants compared to controls as determined through an HPLC-triple TOF-MS/MS. The study reported a significant increase in phenolic acids, flavonoids, and iridoids, which are closer to the antioxidant capacity of plants. The above-mentioned studies are summarized in Table 1.

Furthermore, a correlation between flavonoids and salt stress was reported in sugar beetroot [91]. Using mass spectrometry coupled with UHPLC, the authors found significant increases in flavonoids such as Apigenin-7-glucoside and luteolin in plants under salt stress. The study concluded that the accumulation of Apigenin-7-glucoside and luteolin, with other metabolites such as ascorbic acid may be involved in the salt stress response of plants. An extensive review of metabolomics studies, from metabolomics reprogramming to exogenous metabolite treatments in enhancing plant salt tolerance, was recently published by Patel et al. [92]. The review comprehensively outlines the pivotal functions of the metabolites and metabolic pathways of primary and secondary metabolites in salt-stress tolerance; in addition, it outlines current advances in metabolic engineering in combination with other omics techniques for regulating plant responses and protection against salinity. Razzaq and colleagues [24] described the workflows in plant metabolomics studies to discover and identify metabolic biomarkers and elucidate biotic-stress resistance and abiotic-stress tolerance mechanisms in plants. Thus far, the current review highlights the pivotal roles that metabolomics technologies and associated chemometrics (informatics) tools play in agricultural biotechnology. The study further reviews the applications of the technologies for crop improvement, including biomarker identification for growth, development, and plant protection against biotic and abiotic stress, to assist in plant breeding strategies, as briefly discussed below.

## 4. Metabolomics-Assisted Breeding for Crop Improvement

Given the increasing demand for high-yield and high-productivity crop varieties under unfavourable climatic conditions and the emergence of new and more virulent strains of pathogens, conventional breeding methods are unlikely to meet the growing demand for food. The application of plant metabolomics and the relevant computational tools discussed above have propelled metabolomics sciences as a new frontier for advanced research in plant biotechnology [15,77], particularly plant breeding. The science offers advanced high-throughput screening processes and reduced run-time in metabolic engineering to develop elite crop varieties with improved stress resistance and tolerance, as well as increased crop productivity and yields. Additionally, scientists can now discover and link gene–environment interactions and carry out organism phenotyping and characterization, and metabolic marker identification. These capabilities allow for the application of metabolomics technologies to decipher metabolic networks associated with stress resistance and tolerance, further providing efficient crop-screening opportunities for favourable traits using metabolomics-assisted breeding [93]. The holistic integration of metabolomics with related omics techniques such as transcriptomics, genomics, and proteomics can further open the door to developing strategies to solve unexplored essential agronomic performances. Researchers have applied advanced analytical techniques such as GC/LC, often coupled with MS, the non-destructive NMR spectroscopy, with the aid of chemometrics and chemoinformatics tools; this enables efficient, accurate, and robust metabolic profiling, as wells as metabolite identification and biomarker discovery.

According to Agarwal and Nair [94], traditional scientists and conventional breeders have focused on the characterization of single metabolic features shown to have essential benefits to a plant, such as the carotenoid content of tomatoes, protein content of maize [95] and starch content of potatoes [96], to select favourable varieties for breeding. However, the recent advancements in plant metabolic profiling and the characterization of plant metabolomes have provided great insights into the metabolic and chemical composition of crops such as wheat [97], oat [53], and barley [98], which have potential for metabolic engineering towards crop improvement strategies (methodologies). For instance, in a previous UHPL-QTOF-based cultivar classification study, Mashabela et al. [97] found that wheat cultivars resistant to stripe rust had a higher accumulation of phenolic compounds such as flavonoids and phenylpropanoids compared to their susceptible counterparts. These findings can be used to breed plants with higher flavonoid content by enhancing the expression of the gene responsible for flavonoid biosynthesis—for an improved defense response in crops—as proof of concept for metabolomics–genomics integration. Plant metabolic profiling also provides insights into the regulatory networks responsible for metabolic pathways. This valuable information can form the foundation for genetic-engineering techniques for the modification of metabolic pathways and overall metabolism to suit a plant’s metabolic requirements, either geared towards growth, development and yields, or defense response, in a process known as metabolic engineering [94]. This approach involves two main aspects: the integration of well-established genomics technology and the thriving evolution of metabolomics sciences. The integrated genomics–metabolomics perspective provides relief from the complexity of deducing inter-omics crosstalk without evidence, by offering a tool to monitor the real-time effects of genetic modifications through the subsequent metabolic responses to environmental conditions; thus, it provides a direct link between metabolic and genetic markers associated with favourable traits and is essential for breeding strategies. Furthermore, such markers can be promising candidates for diagnostic or predictive tools for plant breeding [99].

The recent shift to integrated metabolomics and post-genomics studies has enabled scientists to elucidate the gene–metabolite–trait association with the application of techniques such as metabolic quantitative trait loci (mQTLs) and metabolic genome-wide associated studies (mGWAS); these are essential in mapping genetic candidates for plant adaptability to environmental conditions, a vital tool in modern metabolomics-based strategies for plant breeding and crop improvement. Metabolomics offers a crucial bridge in the omics spectrum, linking the phenotype and the genotype (genes encoding quantitative trait loci, also known as QTLs). QTLs are genomic regions on chromosomes that are associated with specified phenotypic traits. The analysis or mapping of QTLs through genome-wide associated studies (GWAS) can reveal the function as part of the perception, regulatory, metabolic, or transduction pathways [25,100]. According to Gong et al. (2013), metabolomics-based GWAS (mGWAS), and QTL (mQTLs) are robust tools for the detection and quantitative analysis of genetic variations associated with metabolic and subsequent phenotypic traits. mQTL analysis assists in identifying candidate genes regulating the biosynthesis of secondary metabolites and provides a comprehensive insight into quantitative genetics through integrated gene-expression analysis and metabolic profiling.

Therefore, the combination of metabolomics studies (GC/LC-MS) and mQTLs has the potential to uncover the genes responsible for observed metabolic perturbations or reprogramming in crops due to changes in environmental conditions. These combined strategies enable researchers to pinpoint the accumulation of tissue-specific secondary metabolites [101] and evaluate stress-responsive metabolites such as sinapic acid, ferulic, and flavones, which serve as antioxidants under stress conditions [102]. A large-scale UPLC-MS-mQTL analysis by Alseekh and colleagues [103] to investigate genomic regions associated with secondary metabolism in tomato fruit pericarp uncovered 679 mQTLs linked to environmental-stress tolerance. The variations in the metabolic adaptations of barley to heat and drought stress were determined via the tentative identification of mQTLs. LC-ion chromatography–tandem MS (IC-MS/MS)-based metabolite profiling revealed mQTLs on genes encoding the enzymatic pathways producing the antioxidants leafy tocopherol, succinate, and glutathione under drought and combined heat-and-drought stress [104]. Furthermore, the application of mQTL analysis in plant–microbe interactions has been reported. The identification and mapping of mQTLs, thus, also have the potential to decipher the regulatory pathways involved in biotic-stress resistance and the genes associated with host–pathogen interactions [24].

Moreover, researchers have applied GWAS to associate specific genetic variations with traits or diseases [105]. In metabolomics studies, mGWAS has been employed to derive links between the biochemical space of plants (plant metabolism) and the genetic variations, and to identify the novel genetic candidates responsible for the metabolic responses of plants to specific environmental conditions or genes encoding specified favourable agronomic traits in crops. As summarised in the studies above, the prospects of linking metabolic events in the plant directly to agronomic traits and candidate genes provide new opportunities for metabolomics-assisted breeding and crop improvement. However, some challenges remain, preventing researchers from realizing the full power and potential of metabolomics technologies. Metabolite annotation is the primary goal of (untargeted) metabolomics studies.

Nevertheless, the complexity of the highly diverse plant metabolome makes metabolite annotation a bottleneck in metabolomics studies, thus limiting the prospects for holistic coverage of the whole plant metabolome. Additionally, the lack of standardized plant metabolomics techniques and workflows makes acquired data highly variable between laboratories, which can render some curated metabolomics data unreliable thus leading to cross-referencing of data within several databases. A tedious and time-consuming task is still advisable. Furthermore, metabolomics studies generate enormous amounts of data at once, and extracting practical information from raw data is a significant challenge. Only a limited number of chemically characterized metabolites exist in data storage databases, while many more have not yet been structurally classified or characterized. Many detected metabolites from untargeted metabolomics studies remain as unknown, unidentified spectral peaks. This indicates the long journey the field of metabolomics and metabolomics technology is yet to undertake, still presenting more open opportunities for growth and development.

## 5. Conclusions and Future Perspectives

In the past two decades, significant advances in metabolomics research have been made due to maturation in technological advances. Achievements came in the form of more advanced analytical tools with higher sensitivity, selectivity and robustness, and the development of reliable computational and chemometrics tools for high-throughput data mining, processing, and statistical analysis. Additionally, the development of chemoinformatics databases has revolutionized the curation and dissemination of metabolomics data, particularly annotated metabolites, and their relevant functional properties, as well as specified roles in metabolic pathways. On the other hand, integrating metabolomics techniques and other omics technologies such as mGWAS and mQTL provides powerful and practical tools for the complete elucidation of the gene–metabolite–trait association and systems biology. It helps to decipher the underlying regulatory mechanism in plant responses to biotic and abiotic stress. This advancement is expected to further revolutionize and broaden the reach of metabolomics studies, particularly in agricultural biotechnology for crop improvement. Moreover, metabolomics applications can assist in identifying the metabolic biomarkers responsible for plant adaptation to environmental conditions, the safety assessment of genetically modified crops, and the elucidation of metabolites associated with nutritional-value crops and fruits for human health. Further exploration of these applications can advance research, and is essential for maintaining global food security and meeting the growing demand for crop productivity and yields.

## Figures and Tables

**Table 1:** Recent exemplary studies showing adaptations of plants during biotic and abiotic stress.

Biotic Stress			
Method	Plant	Summary of Study	Ref.
UHPLC-ESI-MS; q-TOF-MS	*Solanum lycopersicum*	Elevated and concentrated levels of potential biomarkers or stress-signaling molecules were seen during R infection, providing insight into the underlying association of metabolites and defense.	[35]
UHPLC–MS; UHPLC–QqQ-MS	*Solanum lycopersicum*	Time-dependent metabolic changes and tissue-specific reprogramming were observed in response to *Phytophthora capsica* infection.	[36]
UHPLC-ESI-qTOF-MS; UHPLC-QqQ-MS	*Solanum lycopersicum*	Differential reprogramming of amino acids and phytohormones were observed in primary metabolism in response to *Phytophthora capsica* infection.	[72]
^1^H NMR; 2D TOCSY; HSQC	*Triticum aestivum*	Showed that the elevated changes taking place in the host metabolic profile were dependent on wheat inoculated with *Fusarium graminearum* both at ambient and increased CO_2_ levels.	[33]
NMR; GC/LC-MS/MS	*Oryza sativa* L. cv. Hwacheong	Demonstrated metabolic changes in *Magnaporthe grisea*-induced rice cultivars.	[74]
UHPLC-MS; GC-MS	*Oryza sativa* L.	Primary, carbohydrate, and secondary metabolism form a significant part of rice defense mechanisms against *Chilo suppressalis*.	[75]
LC/TOF/MS; LC/QE/MS	*Triticum turgidum* ssp. *durum*	Activation of defense-related phytohormone, and terpenoid-related and shikimate-mediated secondary metabolism in rice responding to *C. suppressalis* feeding and a significant induction of benzoxazoles in wheat genotypes subjected to aphid feeding.	[76]
**Abiotic Stress**			
UHPLC- qTOF-HDMS	*Zea mays*	Differential accumulations of HCAs, HCA derivatives, and flavonoids in maize plants under drought stress.	[37]
UHPLC-qTOF-MS	*Zea mays*	Significant amino acid reduction was observed in nutrient-starved maize plants in comparison to the control.	[38]
LC–MS and GC–MS	*Solanum lycopersicum* L.	Decreased amino acid levels in the leaves of tomato plants due to nutrient deficiency.	[78]
GC-MS	*Hordeum vulgare* L.	Barley plants experienced increased levels of amino acids, sugars, and organic acids when exposed to drought conditions.	[80]
GC-MS	*Triticum* ssp.	Water and nutrient uptake were metabolically activated in the roots and shoots due to a significant increase in amino acids and sugars caused by exposure to drought stress.	[30]
(QTRAP)-MS	*Dendrobium sinense*	An increase in flavonoids, alkaloids, and phenylpropanoids was recorded under drought stress.	[86]
UHPLC-MS/MS	*Triticum aestivum*	Accumulation of phenolics, alkaloids, and flavonoids in wheat genotypes exposed to drought conditions	[87]
FTMS	*Medicago sativa* and *Medicago arborea*	Secondary metabolites from saponins and hydroxycinnamic acids increased salinity-stress tolerance in Medicago sativa and Medicago arborea species.	[89]
HPLC-triple TOF-MS/MS	*Lonicerae Japonicae* Flos	Differential accumulation of secondary metabolites (phenolic acids, flavonoids, and iridoids) in salt-stressed plants compared to controls.	[90]
UPLC-MS	*Beta vulgaris*	Significant increases in flavonoids (Apigenin-7-glucoside and luteolin) in plants under salt stress.	[91]

## Data Availability

Not applicable.

## Abbreviations

P/MAMPs—pathogen-/microbe-associated molecular patterns; PRRs—pathogen-recognition receptors; P/MTI—P/MAMP-triggered immunity; PRPs—pathogenesis-related proteins; HR—hypersensitive response; ISR/SAR—induced systemic resistance/systemic acquired resistance; ETS—effector-triggered susceptibility.
